# Incomplete Reversibility of Estimated Glomerular Filtration Rate Decline Following Tenofovir Disoproxil Fumarate Exposure

**DOI:** 10.1093/infdis/jiu107

**Published:** 2014-02-28

**Authors:** Sophie Jose, Lisa Hamzah, Lucy J. Campbell, Teresa Hill, Martin Fisher, Clifford Leen, Richard Gilson, John Walsh, Mark Nelson, Phillip Hay, Margaret Johnson, David Chadwick, Dorothea Nitsch, Rachael Jones, Caroline A. Sabin, Frank A. Post, for the UK Collaborative HIV Cohort Study Steering Committee, Jonathan Ainsworth, Jane Anderson, Abdel Babiker, David Chadwick, Valerie Delpech, David Dunn, Martin Fisher, Brian Gazzard, Richard Gilson, Mark Gompels, Phillip Hay, Teresa Hill, Margaret Johnson, Stephen Kegg, Clifford Leen, Mark Nelson, Chloe Orkin, Adrian Palfreeman, Andrew Phillips, Deenan Pillay, Frank Post, Caroline Sabin, Memory Sachikonye, Achim Schwenk, John Walsh, Teresa Hill, Susie Huntington, Sophie Josie, Andrew Phillips, Caroline Sabin, Alicia Thornton, David Dunn, Adam Glabay, C. Orkin, N. Garrett, J. Lynch, J. Hand, C. de Souza, M. Fisher, N. Perry, S. Tilbury, D. Churchill, B. Gazzard, M. Nelson, M. Waxman, D. Asboe, S. Mandalia, V. Delpech, J. Anderson, S. Munshi, H. Korat, M. Poulton, C. Taylor, Z. Gleisner, L. Campbell, Abdel Babiker, David Dunn, Adam Glabay, R. Gilson, N. Brima, I. Williams, A. Schwenk, J. Ainsworth, C. Wood, S. Miller, M. Johnson, M. Youle, F. Lampe, C. Smith, H. Grabowska, C. Chaloner, D. Puradiredja, J. Walsh, J. Weber, F. Ramzan, N. Mackie, A. Winston, C. Leen, A. Wilson, M. Gompels, S. Allan, A. Palfreeman, A. Moore, D. Chadwick, K. Wakeman, Stephen Kegg, Paul Main, Mitchell, Hunter, Memory Sachikonye, Phillip Hay, Mandip Dhillon

**Affiliations:** 1 Research Department of Infection and Population Health, University College London; 2 Kings College Hospital National Health Service (NHS) Foundation Trust and King's College London School of Medicine; 3 Mortimer Market Centre, University College Medical School; 4 Imperial College Healthcare NHS Trust; 5 Chelsea and Westminster NHS Foundation Trust; 6 St George's Healthcare NHS Trust; 7 Royal Free Hampstead NHS Trust; 8 London School of Hygiene and Tropical Medicine, London; 9 Brighton and Sussex University Hospitals NHS Trust, Brighton; 10 The Lothian University Hospitals NHS Trust, Edinburgh; 11 South Tees Hospitals NHS Foundation Trust, Middlesbrough, United Kingdom

**Keywords:** tenofovir, highly active antiretroviral therapy, eGFR, eGFR slopes, renal function, kidney

## Abstract

***Background.*** Tenofovir disoproxil fumarate (TDF) has been linked to renal impairment, but the extent to which this impairment is reversible is unclear. We aimed to investigate the reversibility of renal decline during TDF therapy.

***Methods.*** Cox proportional hazards models assessed factors associated with discontinuing TDF in those with an exposure duration of >6 months. In those who discontinued TDF therapy, linear piecewise regression models estimated glomerular filtration rate (eGFR) slopes before initiation of, during, and after discontinuation of TDF therapy. Factors associated with not achieving eGFR recovery 6 months after discontinuing TDF were assessed using multivariable logistic regression.

***Results.*** We observed declines in the eGFR during TDF exposure (mean slopes, −15.7 mL/minute/1.73 m^2^/year [95% confidence interval {CI}, −20.5 to −10.9] during the first 3 months and −3.1 mL/minute/1.73 m^2^/year [95% CI, −4.6 to −1.7] thereafter) and evidence of eGFR increases following discontinuation of TDF therapy (mean slopes, 12.5 mL/minute/1.73 m^2^/year [95% CI, 8.9–16.1] during the first 3 months and 0.8 mL/minute/1.73 m^2^/year [95% CI, .1–1.5] thereafter). Following TDF discontinuation, 38.6% of patients with a decline in the eGFR did not experience recovery. A higher eGFR at baseline, a lower eGFR after discontinuation of TDF therapy, and more-prolonged exposure to TDF were associated with an increased risk of incomplete recovery 6 months after discontinuation of TDF therapy.

***Conclusions.*** This study shows that a decline in the eGFR during TDF therapy was not fully reversible in one third of patients and suggests that prolonged TDF exposure at a low eGFR should be avoided.

Tenofovir disoproxil fumarate (TDF) is a widely used component of combination antiretroviral therapy (cART) [1–3]. Although clinical trials data indicate a low incidence of serious renal adverse effects [4–6], cohort studies have linked TDF use to a decreasing estimated glomerular filtration rate (eGFR) [7], an accelerated decrease in the eGFR [8], proximal tubular dysfunction [9, 10], proteinuria [11], chronic kidney disease (CKD) [12–14], and increased mortality [15]. Scherzer et al [12] evaluated the effects of TDF exposure on renal outcomes in 10 000 human immunodeficiency virus (HIV)–infected treatment-naive patients and found that each year of cumulative exposure was associated with a 30% increase in the risk of proteinuria, an 11% increase in the risk of a rapid decline in the eGFR (defined as >3 mL/minute/1.73 m^2^/year), and a 33% increase in the risk of developing CKD. The authors suggest that the effect of TDF therapy was not fully reversible after discontinuation.

Studies exploring the reversibility of renal function decline following discontinuation of TDF therapy have focused on individuals stopping because of toxicity [16–20] but were small in size with inconclusive outcomes. Wever et al [20] and Yoshino et al [21] studied predominantly HIV-positive men who discontinued TDF therapy for incident CKD or a low eGFR (median, 48.3 mL/minute/1.73 m^2^ [interquartile range {IQR}, 45.3–54.3]) and found that only 42% of 45 patients recovered their baseline eGFR. The majority of individuals had impaired renal function at the start of TDF exposure, and some continued to experience a decline in the eGFR following discontinuation of TDF therapy. Bonjoch et al [22] looked at individuals with normal renal function at baseline who discontinued treatment because of toxicity and found that the eGFR in 59% returned to normal.

There are few studies in which changes in renal function with TDF therapy were investigated using eGFR slopes, and of those that did, none considered the rate of renal decline before TDF exposure [23]. Fafin et al [24] studied the evolution of eGFR in patients with CKD and observed that TDF exposure was associated with a decline in the eGFR and that longer TDF exposure was associated with a lower eGFR. Kalayjian et al [25] examined eGFR slopes before and after initial cART exposure. Although there remained an overall decline in the eGFR during cART, this was modest and slower than that before cART initiation (0.81 mL/minute/1.73 m^2^/year [95% confidence interval [CI], 0.03–1.59]; *P* = .02). In addition, when eGFR slopes were stratified by regimen, a significant improvement was seen in those for whom TDF was coadministered with a protease inhibitor (PI).

Our aim was to evaluate changes in renal function before initiation of, during, and after discontinuation of TDF therapy, using eGFR slopes in HIV-positive individuals who discontinued TDF therapy. We also examined the extent to which the decline in eGFR associated with TDF was reversible following discontinuation of TDF therapy, accounting for preexisting renal decline, and the factors associated with incomplete eGFR reversibility.

## METHODS

### Patients

Data were obtained from the United Kingdom Collaborative HIV Cohort (UK CHIC) Study, which collates routinely collected data on HIV-positive individuals >16 years age from several of the largest HIV clinics in the United Kingdom [26]. The study was approved by a multicenter research ethics committee and by local ethics committees and does not require informed consent. Data up to December 2010 were available from 9 centers that routinely provided creatinine measurements. Eligible subjects were exposed to TDF for a period of at least 6 months. The first available episode of TDF treatment that lasted >6 months was used. Patients who discontinued TDF therapy were included in the analysis of eGFR slopes and those whose eGFR declined during TDF therapy were analyzed for recovery. For the analysis of eGFR slopes and recovery, individuals were required to have at least 6 months of follow-up and ≥3 serum creatinine measurements before initiation of, during, and after discontinuation of TDF therapy.

### Variable Definitions

Serum creatinine measurements were converted to eGFRs, using the CKD Epidemiology Collaboration equation [27, 28]. When a patient's eGFR slope before initiation of TDF therapy was <0, this indicated a preexisting decline in the eGFR. Recovery was then defined differently for those with and those without evidence of a preexisting decline in the eGFR. For patients in whom there was a preexisting decline, we predicted the eGFR at the time of TDF therapy discontinuation by using the eGFR slope before initiation of TDF therapy and the duration of TDF exposure, and recovery was defined at the first of 2 consecutive eGFRs within 5% of this predicted eGFR. For those without evidence of a decline before TDF exposure, recovery was defined at the first of 2 consecutive eGFRs within 5% of the eGFR at the time of TDF initiation (baseline). Sensitivity analyses allowed for greater within-patient variability by changing this 5% cutoff to 10% and 15%. Anyone who did not experience recovery in the eGFR was deemed to have had an incomplete recovery. A normal eGFR was defined as an eGFR of ≥90 mL/minute/1.73 m^2^/year.

### Statistical Analysis

Factors associated with discontinuation of TDF after 6 months were investigated with Cox proportional hazards models. Time-dependent covariates considered were age, AIDS-defining events, CD4^+^ T-cell count, viral load, ART regimen (nonnucleoside reverse-transcriptase inhibitor [NNRTI] based; PI based, including atazanavir; PI based, not including atazanavir; and other regimens), eGFR, hepatitis B status, and hepatitis C status. Time-independent covariates included sex, ethnicity, route of HIV exposure, previous ART exposure (ART naive, experienced with no prior TDF exposure, and experienced with prior TDF exposure) and calendar year when TDF therapy was started. All covariates with a *P* value of < .1 in the univariable analysis were considered for entry into the multivariable model.

In subjects who discontinued TDF treatment, changes in the eGFR before initiation of, during, and after discontinuation of TDF therapy were investigated. Separate piecewise linear regression models were fitted for each patient to estimate eGFR slopes in the 3 periods. eGFR slopes during and after TDF exposure were split into 2 periods: ≤3 months and >3 months (Supplementary Figure 1). This was done to separate any effect of residual drug exposure and early tubular creatinine secretion from longer-term slope estimates. Slopes were stratified according to baseline eGFRs of <60, 60–89, and ≥90 mL/minute/1.73 m^2^.

Factors associated with incomplete eGFR recovery 6 months after discontinuation of TDF therapy were assessed using logistic regression. Factors considered for inclusion in the model were eGFR at start and discontinuation of TDF therapy, time exposed to TDF, CD4^+^ T-cell count and HIV load at start and discontinuation of TDF therapy, being ART naive at start of TDF therapy, cART regimen (PI vs NNRTI vs other), and demographic characteristics. Covariates considered for entry in the multivariable model were chosen a priori as possible confounders or had a *P* value of < .1 in univariable analyses. Analyses were stratified by baseline eGFR to determine whether starting or stopping TDF with an eGFR within the normal range influenced recovery. Sensitivity analyses considered factors associated with incomplete recovery out to 12 and 24 months. We also assessed recovery in those most likely to have discontinued TDF treatment because of toxicity by excluding those with a detectable viral load at the time TDF therapy was discontinued. All analyses were performed in SAS (version 9.3).

## RESULTS

**Figure 1.**
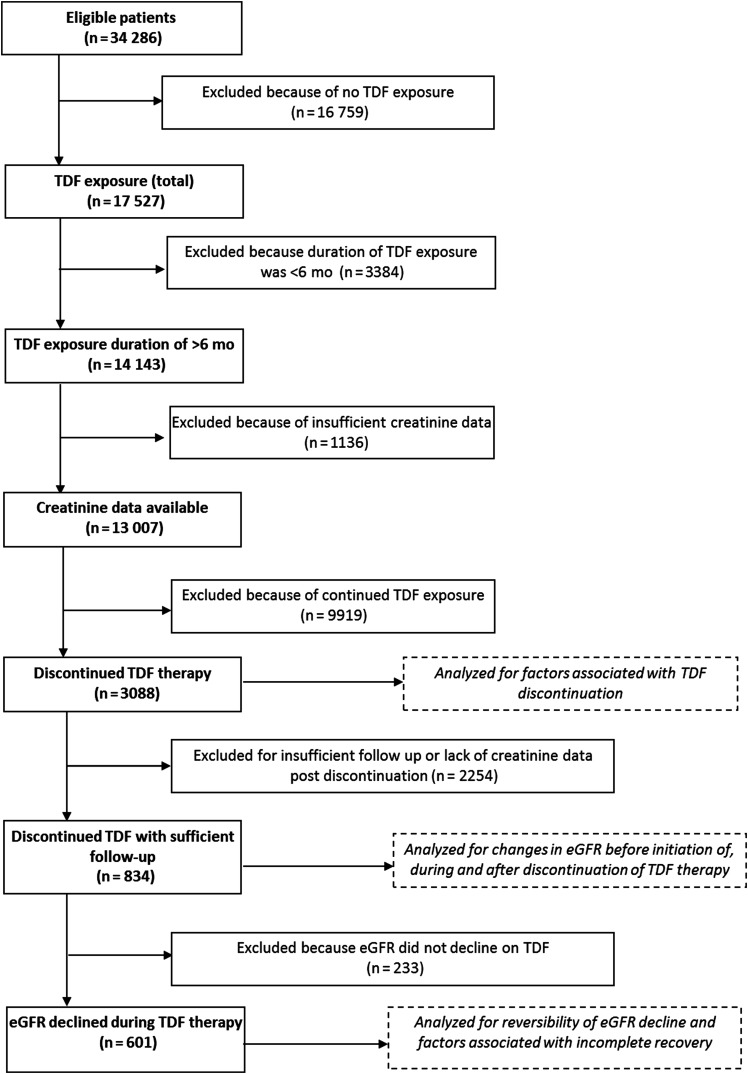
Flow of patient selection for this analysis.

In total, 13 007 were included in the analysis (Figure 1). Baseline characteristics of these individuals are described in Table 1. The majority were white men who acquired HIV through sex with other men; 34.4% were ART naive at the time TDF therapy was started.

**Table 1.** Characteristics of Patients in the United Kingdom Collaborative HIV Cohort Study Who Were Exposed to Tenofovir Disoproxil Fumarate (TDF) for ≥6 Months

Characteristic	Value	
Male sex	10 550	(81.1)
Ethnicity		
White	8300	(63.8)
Black	3026	(23.3)
Other/unknown	1681	(12.9)
Route of HIV exposure		
Homosexual/bisexual sex	8236	(63.3)
Heterosexual sex	3713	(28.6)
IDU	356	(2.7)
Other/unknown	702	(5.4)
Calendar year		
1999–2003	2482	(19.1)
2004–2007	3992	(30.7)
2008–2010	6533	(50.2)
Previous exposure to TDF	603	(4.6)
ART naive	4466	(34.4)
ARV class		
PI based (no ATZ)	2690	(20.7)
PI based (with ATZ)	1329	(10.2)
NNRTI based	7098	(54.6)
Other	1890	(14.5)
Previous AIDS-defining event	3053	(23.5)
HBV status		
Negative	8412	(64.7)
Positive	599	(4.6)
Not tested	3996	(30.7)
HCV status		
Negative	8619	(66.3)
Positive	730	(5.6)
Not tested	3658	(28.1)
eGFR		
<60 mL/min/1.73 m^2^	166	(1.4)
60–74 mL/min/1.73 m^2^	985	(8.5)
75–89 mL/min/1.73 m^2^	2572	(22.1)
≥90 mL/min/1.73 m^2^	7898	(68.0)
Age, y	40	(34–46)
CD4^+^ T-cell count, cells/mm^3^	303	(190–482)
HIV load, log_10_ copies/mL	2.9	(1.7–4.7)

Data are no. (%) of patients or median value (interquartile range) and were recorded at the start of TDF therapy.

Abbreviations: ART, antiretroviral therapy; ARV, antiretroviral; ATZ, atazanavir; eGFR, estimate glomerular filtration rate; HBV, hepatitis B virus; HCV, hepatitis C virus; HIV, human immunodeficiency virus; IDU, injection drug use; PI, protease inhibitor; NNRTI, nonnucleoside reverse-transcriptase inhibitor; NRTI, nucleoside reverse-transcriptase inhibitor.

^a^ Data are for 11 621 patients with eGFR measure available in the 6 months preceding TDF therapy.

### Factors Associated With Discontinuation of TDF Therapy

A total 3088 (23.7%) patients discontinued TDF therapy, for an incidence rate of 7.3 cases/100 person-years (95% CI, 7.0%–7.5%). The median exposure time was 2.6 years (IQR, 1.5–4.8 years). A decline in the eGFR during TDF exposure was experienced by 1882 individuals (61%) who discontinued TDF therapy; 56.3%, 21.0%, 12.3%, and 10.3% discontinued TDF therapy with an eGFR of ≥90, 75–89, 60–74, and <60 mL/minute/1.73 m^2^, respectively, compared with 65.6%, 22.2%, 9.8%, and 2.4% in the same eGFR thresholds at baseline. Of 2906 individuals with a viral load measurement before stopping TDF therapy, 2049 (70.5%) had an undetectable viral load. A higher viral load (hazard ratio [HR], 1.6 per 1 log_10_ increase [95% CI, 1.55–1.66]) and a lower eGFR (eGFR 60–74 mL/minute/1.73 m^2^: HR, 1.21 [95% CI, 1.08–1.25]; eGFR < 60 mL/minute/1.73 m^2^: HR, 3.90 [95% CI, 3.45–4.42]) were associated with an increased risk of TDF discontinuation. Patients with ART experience and no prior TDF exposure were less likely to discontinue TDF therapy, compared with ART-naive individuals (HR, 0.78 [95% CI, .71–.86]), whereas patients with ART experience including previous TDF therapy showed an increased likelihood of discontinuing TDF therapy (HR, 1.24 [95% CI, 1.07–1.4]). A higher CD4^+^ T-cell count (HR, 0.99 per 50 cells/mm^3^ increase [95% CI, .98–.99]), coadministration of TDF with an NNRTI (HR, 0.56 [95% CI, .50–.63]), and a previous AIDS-defining event (HR, 0.83 [95% CI, .77–.90]) were associated with a decreased likelihood of discontinuing TDF therapy (Supplementary Table 1).

### Changes in eGFR Before Initiation of, During, and After Discontinuation of TDF Therapy

Of 3088 patients who discontinued TDF therapy, 834 (27.0%) had sufficient follow-up time and creatinine data to be included in analyses of eGFR slopes. Those included were more likely to be white (*P* < .0001), to be men who have sex with men (*P* < .0001), to have higher CD4^+^ T-cell counts (*P* < .0001), to have lower viral loads (*P* < .0001), to be cART naive at the start of TDF therapy (*P* < .0001), to have started TDF therapy in an earlier year (*P* < .0001), and to have experienced a previous AIDS-defining event (*P* = .0003).

The median follow-up durations before initiation of, during, and after discontinuation of TDF therapy were 5.8 years (IQR, 3.2–7.2 years), 2.4 years (IQR, 1.4–3.9 years), and 2.2 years (IQR, 1.2–3.8 years). Slopes before initiation of, during, and after discontinuation of TDF therapy are given in Table 2. Before TDF exposure, a small decrease in the eGFR of −0.9 mL/minute/1.73 m^2^/year (95% CI, −1.6 to −.2) was seen in all patients, with a steeper decrease of −3.1 mL/minute/1.73 m^2^/year (95% CI, −4.6 to −1.7) during TDF exposure (*P* = .007), compared with mean slopes of −0.2 mL/minute/1.73 m^2^/year (95% CI, −.6 to .3) and 0.3 mL/minute/1.73 m^2^/year (95% CI, .1–.5) before and during TDF exposure, respectively, among 5669 individuals fulfilling the same inclusion criteria who did not discontinue TDF treatment. In patients who discontinued TDF therapy, the mean eGFR increased after discontinuation (0.8 mL/minute/1.73 m^2^/year [95% CI, .1–1.5]). During the first 3 months following initiation and discontinuation, there were steep decreases and increases in mean slopes (−15.7 mL/minute/1.73 m^2^/year [95% CI, −20.5 to −10.9]) and 12.5 mL/minute/1.73 m^2^/year [95%CI, 8.9–16.1], respectively). When slopes were stratified by baseline eGFR, those with an eGFR of <60 mL/minute/1.73 m^2^ had experienced greater decline before TDF exposure. During TDF exposure, they experienced smaller declines in the eGFR in the first 3 months (−2.5 mL/minute/1.73 m^2^/year [95% CI, −17.1 to 12.1]; *P* = .32) compared with those with higher eGFRs and somewhat greater declines after this point (−8.3 mL/minute/1.73 m^2^/year [95% CI, −18.1 to 1.5]; *P* = .43). Recovery appeared greater immediately following discontinuation of TDF therapy (23.8 mL/minute/1.73 m^2^/year [95% CI, 8.5–39.0]; *P* = .025), but there were no differences in long-term slopes after discontinuation.

**Table 2.** Estimated Glomerular Filtration Rate (eGFR) Slopes Before Initiation of, During, and After Discontinuation of Tenofovir Disoproxil Fumarate (TDF) Exposure

Interval, Relative to TDF Exposure	Overall (n = 834)	By Baseline eGFR		
		<60 mL/min/1.73 m^2^ (n = 24)	60–89 mL/min/1.73 m^2^ (n = 322)	≥90 mL/min/1.73 m^2^ (n = 477)
Before initiation	−0.9 (−1.6 to −0.2)	−5.2 (−8.4 to −2.0)	−1.4 (−2.2 to −0.5)	−0.4 (−1.5 to 0.6)
During				
≤3 mo	−15.7 (−20.5 to −10.9)	−2.5 (−17.1 to 12.1)	−16.9 (−27.0 to −6.8)	−15.3 (−19.9 to −10.7)
>3 mo	−3.1 (−4.6 to −1.7)	−8.3 (−18.1 to 1.5)	−2.6 (−5.5 to 0.4)	−3.3 (−4.8 to −1.8)
After discontinuation				
≤3 mo	12.5 (8.9–16.1)	23.8 (8.5–39.0)	15.8 (10.0–21.6)	9.5 (4.7–14.3)
>3 mo	0.8 (0.1–1.5)	−0.4 (−6.4 to 5.7)	0.3 (−0.8 to 1.4)	1.2 (0.12–2.1)

Data are mean values (95% confidence intervals) from a piecewise linear regression model.

### eGFR Recovery

**Figure 2.**
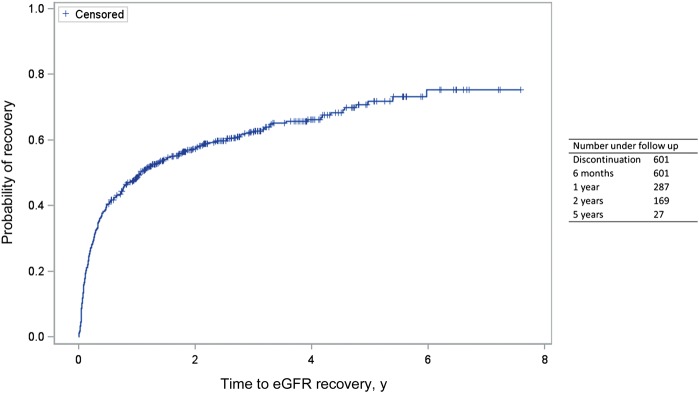
Kaplan-Meier plot showing the cumulative proportion of individuals discontinuing tenofovir disoproxil fumarate (TDF) therapy following a decline in the estimated glomerular filtration rate (eGFR) that experienced eGFR recovery following discontinuation.

Of 834 patients who discontinued TDF therapy with sufficient follow-up to assess recovery, 601 (72.1%) experienced a decline in the eGFR during TDF exposure. Median eGFRs at start and stop of TDF therapy were 94 mL/minute/1.73 m^2^ (IQR, 81–108) and 77 mL/minute/1.73 m^2^ (IQR, 57–94), respectively. A total of 85 (27.1%) of 314 patients with and 147 (51.2%) of 287 without a preexisting decline in the eGFR did not experience a recovery in the GFR after discontinuing TDF therapy. For patients in whom eGFR recovery was incomplete, 45 (20.1%), 40 (17.9%), 54 (24.1%), and 85 (37.9%) had an eGFR of ≥90, 75–89, 60–74, and <60 mL/minute/1.73 m^2^ at the time TDF therapy was discontinued (8 had an unknown eGFR at discontinuation). The median time to recovery was 1.3 years (95% CI, 1.0–1.9) after discontinuation of TDF therapy, but recovery may have continued out to 5 years (Figure 2). The median eGFR was significantly higher at baseline (97 mL/minute/1.73 m^2^ [IQR, 86–110] vs 92 mL/minute/1.73 m^2^ [IQR, 77–106]; *P* ≤ .0001) and lower at TDF therapy discontinuation (66 mL/minute/1.73 m^2^ [IQR, 52–86] vs 82 mL/minute/1.73 m^2^ [IQR, 64–97]; *P* < .0001) in patients with incomplete recovery. A total of 150 individuals (25.0%) were not receiving cART immediately following discontinuing TDF therapy, including 22.4% with incomplete recovery of the eGFR and 26.6% with full recovery the eGFR. Approximately 59% of those who either did or did not have complete recovery of the eGFR were receiving a regimen containing a PI following discontinuation of TDF therapy (*P* = .95), with approximately 16% in both groups receiving atazanavir (*P* = .99). Whereas 21% of those who experienced eGFR recovery received a NNRTI as part of their cART regimen, 31% of those in whom the eGFR did not recover received a NNRTI (*P* = .006).

A lower eGFR at discontinuation of TDF therapy and a higher eGFR at initiation of TDF therapy were independently associated with an increased odds of incomplete recovery at 6 months (Table 3). Longer TDF exposure was also associated with an increased odds of incomplete recovery. Receiving a PI-based regimen (vs an NNRTI-based regimen) at the start of the TDF episode was associated with a decreased odds of experiencing incomplete recovery. Similar results were obtained when recovery was assessed at 12 or 24 months and when stratified by baseline eGFR (ie, < 90 vs ≥90 mL/minute/1.73 m^2^; data not shown).

**Table 3.** Results of Univariable and Multivariable Logistic Regression Modeling to Determine Factors Associated With Incomplete Recovery of the Estimated Glomerular Filtration Rate (eGFR) 6 Months After Discontinuation of Disoproxil Fumarate (TDF)

Factor	Univariable			Multivariable		
	OR	(95% CI)	*P*	OR	95% CI	*P*
Age at initiation of TDF therapy (per 10 y increase)	1.13	(.95–1.34)	.17	1.00	(.80–1.24)	.57
Sex						
Male	1.00			1.00		
Female	0.67	(.43–1.03)	.070	0.95	(.52–1.74)	.87
Ethnicity						
White	1.00			1.00		
Black	0.59	(.38–.92)	.020	0.82	(.45–1.52)	.53
Other/unknown	1.00	(.57–1.76)	.99	1.06	(.57–1.96)	.85
Route of HIV exposure						
Homosexual/bisexual sex	1.00					
Heterosexual sex	0.75	(.51–1.10)	.14	…		
IDU	0.63	(.29–1.34)	.29	…		
Other/unknown	1.00	(.32–3.11)	1.00	…		
ART naive at initiation of TDF therapy						
No	1.00			…		
Yes	0.93	(.55–1.57)	.78	…		
Regimen class at initiation of TDF therapy						
NNRTI	1.00			1.00		
PI	0.70	(.48–1.02)	.066	0.60	(.39–.91)	.018
Other	0.75	(.49–1.14)	.18	0.69	(.43–1.11)	.13
CD4 count at TDF stop (per 50 cells/mm^3^ increase)	1.01	(.98–1.05)	.37	…		
Undetectable viral load at discontinuation of TDF therapy						
No	1.00			1.00		
Yes	1.39	(.93–2.04)	.087	0.96	(.63–1.48)	.86
eGFR						
At initiation of TDF therapy						
≥90 mL/min/1.73 m^2^	1.00			1.00		
75–90 mL/min/1.73 m^2^	1.10	(.74–1.64)	.65	0.47	(.28–.78)	.004
60–74 mL/min/1.73 m^2^	0.61	(.38–.99)	.046	0.14	(.07–.27)	<.0001
<60 mL/min/1.73 m^2^	0.24	(.08–.69)	.008	0.04	(.01–.15)	<.0001
At discontinuation of TDF therapy						
≥90 mL/min/1.73 m^2^	1.00			1.00		
75–90 mL/min/1.73 m^2^	1.59	(1.02–2.51)	.043	2.18	(1.31–3.62)	.003
60–74 mL/min/1.73 m^2^	2.38	(1.49–3.80)	.0003	4.81	(2.61–8.89)	<.0001
<60 mL/min/1.73 m^2^	2.96	(1.89–4.65)	<.0001	13.18	(6.29–27.6)	<.0001
Duration of TDF therapy (per year increase)	1.20	(1.09–1.32)	.0003	1.15	(1.03–1.28)	.012

Abbreviations: ART, antiretroviral therapy; CI, confidence interval; HIV, human immunodeficiency virus; IDU, injection drug use; OR, odds ratio; NNRTI, nonnucleoside reverse-transcriptase inhibitor; NRTI, nucleoside reverse-transcriptase inhibitor; PI, protease inhibitor.

In 580 patients who discontinued TDF therapy with an undetectable viral load, mean eGFR slopes before initiation of, during, and after discontinuation of TDF therapy were similar to those seen in the whole group (−0.5 mL/minute/1.73 m^2^/year before initiation of therapy [95% CI −1.2 to .1]), −3.4 mL/minute/1.73 m^2^/year during therapy [95% CI, −5.2 to −1.6], and 0.9 mL/minute/1.73 m^2^/year after discontinuation of therapy [95% CI, .2–1.7]) and 41.2% of those with a decline in the eGFR did not recover the eGFR during follow-up. Factors associated with increased likelihood of incomplete recovery in this group were similar to those described above; higher eGFR at TDF initiation, lower eGFR at TDF discontinuation and longer time on TDF. Reduced odds of incomplete recovery were again observed when starting a PI-based regimen with TDF (data not shown). Varying the choice of cutoff for defining recovery to 10% and 15% meant that only 27.8% and 7% of individuals, respectively, experienced incomplete eGFR recovery. At a 10% cutoff, the factors associated with incomplete recovery remained unchanged. At a 15% cutoff, only eGFRs at initiation and discontinuation of TDF therapy were associated with incomplete recovery at 6 months (results not shown).

## DISCUSSION

In this large cohort of predominately white, HIV-infected men, approximately one quarter of patients discontinued TDF therapy after an exposure duration of at least 6 months. An accelerated decline in the eGFR was observed during TDF exposure, with substantial recovery in the first 3 months after discontinuation of TDF. Nonetheless, 38% of patients did not experience a recovery in the eGFR to within 5% of the baseline eGFR. An eGFR of <75 mL/minute/1.73 m^2^ at the start of TDF therapy was associated with an increased risk of discontinuing TDF therapy, whereas an eGFR of <90 mL/minute/1.73 m^2^ at the time of discontinuation was associated with an increased risk of incomplete reversibility, as was longer exposure to TDF. These data support renal function monitoring before and during TDF exposure, and they caution against continued TDF exposure in patient with or approaching CKD.

Underlying mechanisms of TDF toxicity have not been fully elucidated. Tenofovir is renally excreted by both glomerular filtration and active tubular secretion. In the proximal renal tubules, TDF is transported across the basolateral membrane via human organic ion transporters 1 and 3 [29] and across the apical membrane via multidrug-resistant protein 2 (MRP2) and MRP4 [30]. Tenofovir toxicity has been linked to an increased plasma drug concentration [31], and therefore mechanisms that interfere with tenofovir excretion may increase the risk of toxicity. A low GFR will cause impaired TDF filtration; the resulting increased plasma concentrations will promote active tubular excretion. Polymorphisms in genes such as ABCC4 (which encodes MRP4), ABCC2 (which encodes MRP2), and ABCC10 (which encodes MRP7) are thought to lead to altered TDF handling and intracellular accumulation of TDF [32]. Increased intracellular TDF concentrations are postulated to cause mitochondrial toxicity, with features such as enlargement, depletion, and dysmorphic mitochondrial changes seen in severe cases of TDF-induced proximal tubulopathy [33]. Increases in TDF plasma levels of approximately 20%–30% may also occur during coadministration of TDF with a boosted PI [34]. Clinically, this combination has been associated with worse renal outcomes than TDF and a non-PI containing regimen [35–37]. Proposed mechanisms include increased absorption of TDF via PI-related inhibition of P-glycoprotein [38] or ritonavir-inhibited secretion of TDF via MRP2 [39].

We observed an accelerated decline in the eGFR during TDF therapy in all strata of eGFRs, a phenomenon seen in other studies [40, 41]. The rapid changes in eGFR seen in the 3-month period between the start and discontinuation of TDF therapy has been noted previously [21, 41]. In part, this may be explained by residual tenofovir exposure or coadministered drugs inhibiting tubular creatinine excretion, although we were unable to investigate this in the current study. When the actual GFR was measured in a study of individuals receiving cART who switched to TDF-based regimens, although the eGFR declined, there was no change in the measured GFR [42]. Beyond the initial 3 months, the average overall decline in eGFR was modest (−3.1 mL/minute/1.73 m^2^/year). This is consistent with previous studies that suggested that the clinical magnitude of TDF-related renal decline was limited. A meta-analysis of TDF-containing regimens versus non–TDF-containing regimens demonstrated a mean difference in creatinine clearance between the 2 groups of only 3.92 mL/min (95% CI, 2.13–5.70) [40], and similarly, in a cohort study with a 10-year follow-up duration, the cumulative eGFR loss attributable to TDF after 4 years was only −3.09 mL/min/1.73 m^2^ [41] but slightly greater than the decline seen within the large US cohort (84% of whom were receiving TDF-based regimens) of −1.37 mL/min/1.73 m^2^ (95% CI, −2.02–.72) [25].

Adverse effects of tenofovir on the kidney are likely the result of tubular injury. Following ischemic or toxic insult, renal tubular cells may undergo some recovery with reversibility of renal tubular damage as demonstrated in animal models [31] and in observational human studies [43]. In our cohort, approximately 40% of patients with a decline in the eGFR had incomplete eGFR recovery after discontinuation of TDF therapy, the majority (62%) of whom had an eGFR of <75 mL/minute/1.73 m^2^ when TDF therapy was discontinued. Persistently impaired renal function following TDF discontinuation may reflect irreversible tenofovir-induced kidney damage or progression of underlying CKD. Because the eGFR at the time TDF therapy was discontinued was an important predictor of incomplete recovery, the benefits of continued TDF exposure should be reviewed in patients with an eGFR of <90 mL/minute/1.73 m^2^, and discontinuation of TDF therapy should be considered before the eGFR decreases to <60 mL/minute/1.73 m^2^ (ie, those with a declining eGFR in the range of 60 to 75 mL/minute/1.73 m^2^).

We were surprised to note that a lower eGFR at the start of TDF therapy was protective against incomplete recovery. Although only small numbers of patients started with an eGFR of < 60 mL/minute/1.73 m^2^, the trend toward a better recovery with a lower eGFR suggests a real phenomenon. This may reflect the findings in the Development of Antiretroviral Therapy (DART) trial, in which those with a lower eGFR at baseline had the greatest increase after starting treatment [44]. Alternatively, low eGFRs have been linked to increased tenofovir concentrations [45], and therefore withdrawal of a higher tenofovir concentration may allow better eGFR recovery. We feel it unlikely to be due to a low threshold for discontinuation in individuals with a decreased eGFR at initiation of TDF therapy, because on the duration of TDF therapy was taken into account in the analysis.

We observed higher levels of eGFR recovery following discontinuation of TDF therapy than those observed in previous studies [20, 21]. This may be because of our definition of recovery (which considered reductions of up to 5% from baseline); because we included discontinuations for any cause, not just suspected TDF toxicity; and because we took into account a decline in the eGFR before TDF initiation. Using at least 2 consecutive values to define recovery suggests that our findings are robust and that recovery of the eGFR following discontinuation of TDF therapy is achievable in the majority of patients. Although recovery was not complete in all cases, it has previously been reported to continue out to 5–17 months [20–22]. We saw recovery up to 5 years out, which may reflect the longer follow-up time available. Factors previously associated with greater improvements in eGFR following discontinuation of TDF therapy included concomitant PI use [20], which was postulated to have been due to the withdrawal of TDF at a higher tenofovir plasma concentration; rapid decline of eGFR within the first month of TDF exposure [21]; and higher nadir and discontinuation CD4^+^ T-cell counts [22]. In our cohort, we were unable to replicate these findings, with the exception of PI use. The latter may have occurred because some patients who discontinued TDF therapy switched from an NNRTI to a ritonavir-boosted PI, resulting in enhanced inhibition of MATE-1–mediated tubular creatinine secretion [46].

Strengths of this study include the use of a large HIV-positive cohort; inclusion of patients who discontinued TDF therapy for any reason, not just toxicity; and a prolonged follow-up duration. We took into account renal declines detected before initiation of TDF therapy when assessing the reversibility of renal decline and the variability of eGFR, allowing a 5% change from baseline. The 5% change was intended to account for intraindividual variance in creatinine (reported to be between 4.2%–14.4% [47–49]), intraanalytic variance, and, therefore, the calculated coefficient of variance for MDRD and CKD-Epi (4.7% [48] and 7.2% [50], respectively). Using a conservative estimate of variability of 5%, we found that the majority of patients had a recovered eGFR, and sensitivity analysis allowing for 10% and 15% variability unsurprisingly yielded further improvements in recovery rates.

A limitation of this study is the variable frequency of eGFR measurements in this observational setting, with creatinine data unavailable for a substantive section of the cohort. Infrequent eGFR measurements will impact eGFR slopes estimated by linear regression. However, use of a mixed-effects regression model to account for within-subject variability and correlated data produced very similar results. The mean eGFR slope estimates before initiation of, during, and after discontinuation of TDF therapy, according to mixed-effects models, were −0.4 mL/minute/1.73 m^2^/year (95% CI, −.6 to −.2), −3.5 mL/minute/1.73 m^2^/year (95% CI, −4.1 to −2.9), and 0.3 mL/minute/1.73 m^2^/year (95% CI, −.0 to .6). A total of 262 individuals (43.6%) did not have a recovery in the eGFR during follow-up, compared with 38.6% according to linear regression models.

We were unable to consider other factors associated with discontinuation of TDF therapy, such as nonadherence or HIV drug resistance, or factors that may be associated with recovery, such as cardiovascular and renal risk factors (diabetes, hypertension, and proteinuria), a reliable indicator of muscle mass, clinical and socioeconomic status, and the impact of loss to follow-up. We cannot exclude the possibility that any observed renal decline was due to other drugs, rather than to TDF. The length of follow-up may not have allowed for maximum renal recovery, and the lack of access to individual patient records meant that those who stopped for renal toxicity could not be defined. However, when considering only those with an undetectable viral load at discontinuation of TDF therapy, we did not see any difference in our results.

In conclusion, for the majority of patients who discontinue TDF therapy, recovery of the eGFR is achievable. Patients with CKD who initiated TDF therapy were at risk of a further decline in the eGFR, whereas ongoing TDF exposure increased the risk of an incomplete eGFR recovery. This study supports continued renal monitoring during exposure to TDF and cautions against prolonged TDF exposure in individuals with a declining eGFR.

## STUDY GROUP MEMBERS

The UK CHIC Steering Committee consists of Jonathan Ainsworth, Jane Anderson, Abdel Babiker, David Chadwick, Valerie Delpech, David Dunn, Martin Fisher, Brian Gazzard, Richard Gilson, Mark Gompels, Phillip Hay, Teresa Hill, Margaret Johnson, Stephen Kegg, Clifford Leen, Mark Nelson, Chloe Orkin, Adrian Palfreeman, Andrew Phillips, Deenan Pillay, Frank Post, Caroline Sabin (principle investigator), Memory Sachikonye, Achim Schwenk, and John Walsh.

Central Coordination was performed at the UCL Research Department of Infection and Population Health, Royal Free Campus, London (by Teresa Hill, Susie Huntington, Sophie Josie, Andrew Phillips, Caroline Sabin, and Alicia Thornton), and the Medical Research Council Clinical Trials Unit, London (by David Dunn and Adam Glabay).

Participating centers (and investigators) comprise Barts and The London National Health Service (NHS) Trust, London (C. Orkin, N. Garrett, J. Lynch, J. Hand, and C. de Souza); Brighton and Sussex University Hospitals NHS Trust (M. Fisher, N. Perry, S. Tilbury, and D. Churchill); Chelsea and Westminster Hospital NHS Trust, London (B. Gazzard, M. Nelson, M. Waxman, D. Asboe, and S. Mandalia); Health Protection Agency–Centre for Infections London (V. Delpech); Homerton University Hospital NHS Trust, London (J. Anderson and S. Munshi); King's College Hospital NHS Foundation Trust, London (H. Korat, M. Poulton, C. Taylor, Z. Gleisner, and L. Campbell); *Medical Research Council Clinical Trials Unit (MRC CTU)*, London (Abdel Babiker, David Dunn, Adam Glabay); Mortimer Market Centre, London (R. Gilson, N. Brima, and I. Williams); North Middlesex University Hospital NHS Trust, London (A. Schwenk, J. Ainsworth, C. Wood, and S. Miller); Royal Free NHS Trust and UCL Medical School, London (M. Johnson, M. Youle, F. Lampe, C. Smith, H. Grabowska, C. Chaloner, and D. Puradiredja); Imperial College Healthcare NHS Trust, London (J. Walsh, J. Weber, F. Ramzan, N. Mackie, and A. Winston); The Lothian University Hospitals NHS Trust, Edinburgh (C. Leen and A. Wilson); North Bristol NHS Trust (M. Gompels and S. Allan); University of Leicester NHS Trust (A. Palfreeman and A. Moore); South Tees Hospitals NHS Foundation Trust (D. Chadwick and K. Wakeman); *Woolwich, South London Healthcare NHS Trust* (Stephen Kegg, Paul Main, Dr. Mitchell, Dr. Hunter), *UK Community Advisory Board* (Memory Sachikonye); *St. George's Healthcare NHS Trust* (Phillip Hay, Mandip Dhillon).

## Supplementary Data

Supplementary materials are available at *The Journal of Infectious Diseases* online (http://jid.oxfordjournals.org/). Supplementary materials consist of data provided by the author that are published to benefit the reader. The posted materials are not copyedited. The contents of all supplementary data are the sole responsibility of the authors. Questions or messages regarding errors should be addressed to the author.

Supplementary Data
