# Supplementary material for: Incomplete Reversibility of Estimated Glomerular Filtration Rate Decline Following Tenofovir Disoproxil Fumarate Exposure
Source: J Infect Dis. 2014 Feb 28;210(3):363–73. doi: 10.1093/infdis/jiu107 (PMC4091582; doi:10.1093/infdis/jiu107)
Supplement: Supplementary Data [file supp_210_3_363__index.html]

Incomplete Reversibility of Estimated Glomerular Filtration Rate Decline Following Tenofovir Disoproxil Fumarate Exposure — Incomplete Reversibility of Estimated Glomerular Filtration Rate Decline Following Tenofovir Disoproxil Fumarate Exposure — Supplementary Data 

# Incomplete Reversibility of Estimated Glomerular Filtration Rate Decline Following Tenofovir Disoproxil Fumarate Exposure
